# Parental perceptions of a wearable vital signs monitor for observation of newborns: in-depth interviews in three tertiary hospitals, Southwest Nigeria

**DOI:** 10.3389/fdgth.2025.1597651

**Published:** 2025-07-28

**Authors:** Yetunde Olufisayo John-Akinola, Adejumoke Idowu Ayede, Ayomide Adeyemi, Assumpta Nantume, Adesewa Oluwatomisin Olaleye, Olugbenga Akinrinoye, Olukemi Oluwatoyin Tongo, Michael Abe Alao, Adenike Oluleye, Oluseye Sobande, Olatayo Sunday Olayemi, Bertha Akinyi Oketch, Rosena Olubanke Oluwafemi, Patricia Ireti Eniowo, Samuel Ojo, Omolayo Adebukola Olubosede, Emmanuel Olaseinde Bello, Ismaila Sani, Teresa Cauvel, Sona Shah

**Affiliations:** ^1^ College of Medicine, University of Ibadan and University College Hospital, Ibadan, Nigeria; ^2^ Neopenda, PBC, Chicago, IL, United States; ^3^ Mother and Child Hospital Akure, Akure, Ondo State, Nigeria; ^4^ University of Medical Sciences Teaching Hospital Complex, Akure, Ondo State, Nigeria

**Keywords:** parental perceptions, wearable vital signs monitor, newborns, acceptability, satisfaction

## Abstract

**Introduction:**

The first 28 days of a newborn's life are a critical time for their survival and subsequent growth and development. Wearable devices have emerged as a potential solution for clinical monitoring, offering affordability, accessibility, and improved patient care. This study explored the acceptability, satisfaction, and perceived benefits of the neoGuard wearable vital signs monitoring device among parents/guardians of sick newborns in Nigeria.

**Methods:**

This was a qualitative study conducted between April and December 2022 at three tertiary level health facilities in Nigeria. In-depth interviews were conducted with 17 parents/guardians whose newborns were monitored using the neoGuard technology. Participants were selected based on specific criteria, including observation of at least 2 h of use of the neoGuard technology on their newborn, and interviews were conducted in the participants’ preferred language. Interviews were moderated with a semi-structured interview guide and audio recorded. Data were transcribed, coded and analyzed thematically using NVivo software.

**Results:**

The majority of participants expressed positive reactions to the neoGuard device, although some initial concerns were reported. Participants appreciated the device's functionality, ease of use, and potential to detect health issues in newborns. The device did not interfere with routine care activities such as cleaning, breastfeeding, or kangaroo care. Participants expressed high levels of confidence in the device's safety and expressed satisfaction with its performance. They suggested improvements such as designing the device to be worn on the wrist instead of the forehead.

**Conclusion:**

Overall, the neoGuard wearable vital signs monitor was well accepted by parents/guardians of sick newborns in Nigeria. The device's functionality, ease of use and potential to improve newborn health were positively perceived. Some suggestions for design improvements were provided. These findings highlight the importance of considering the perspectives of parents/guardians, alongside clinicians and other stakeholders, when implementing new technologies in clinical care. Further research should examine the device's clinical impact and cost-effectiveness while considering the experiences and perceptions of both healthcare providers and patients’ guardians to inform adoption into clinical practice.

## Introduction

The first 28 days of a newborn's life are a critical time for their survival and subsequent growth and development. During this time, newborns face a high risk of death, with an average global rate of 18 deaths per 1,000 live births. In 2021 alone, 2.3 million newborns deaths occurred globally—an equivalent of approximately 6,400 neonatal deaths daily. Sub-Saharan Africa in particular experiences a disproportionate number of neonatal deaths, accounting for 43% of the global neonatal deaths, or 27 deaths per 1,000 live births. Nigeria has the highest number of neonatal deaths in Africa, with approximately 271,000 deaths in 2020 alone, proportional to 35.5 deaths per 1,000 live births (1).

Newborn mortality is largely preventable, as it often stems from treatable diseases and conditions that can be effectively managed with timely interventions and high-quality care from trained healthcare providers. In Nigeria, the leading causes of neonatal death are low birth weight, intrapartum-related complications such as birth asphyxia, and infections like sepsis and pneumonia (1–3), for which effective treatments exist**.** But despite the concerted effort to increase access to comprehensive newborn interventions in low-resource settings, one area in management of critical newborns where low-and-middle-income countries (LMICs) are still significantly lagging is in leveraging sophisticated medical technology like continuous vital signs monitors.

Vital signs monitoring is a usual practice for management of small or sick newborns because it helps to detect early signs of clinical deterioration in real time among susceptible patients, enabling healthcare providers to intervene more quickly with effective treatment when needed. However, due to the prohibitive costs associated with conventional bedside patient monitors, most healthcare providers in LMICs rely on intermittent and manual methods of vital signs monitoring such as digital thermometers to measure temperature, hand-held pulse oximeters to measure pulse rate and oxygen saturation and timers to manually count breaths per minute; all of which are less effective in identifying sudden and rapid changes in a newborn's status. Besides, single-point assessment of these newborns might be unrepresentative due to immature organ systems and nonspecific physiological responses to stimuli or events. The limited capacity for vital signs monitoring therefore puts vulnerable patients in LMICs at a higher risk of delayed medical intervention. This gap in care is further exacerbated by critical shortages in healthcare staff, with countries like Nigeria facing significant challenges in training, retaining and evenly distributing their health workforce to serve an ever growing population (4, 5).

In recent years, the adoption of wearable devices for clinical monitoring has been posited as an affordable and viable solution for various populations and settings. The current literature on observation of patients with wearable devices covers multiple use cases including paediatric and adult patients with pneumonia (6), pregnant women at risk of hypertension and shock (7, 8), post-surgical patients (9–11), survivors of stroke (12) and even unborn (13) and newborn patients (14–16). The emerging trend of wearable technologies in clinical care (17) offers several potential benefits such as increased affordability, promoting health worker efficiency, proactive management and better treatment of various medical conditions (18, 19), as well as lowering the rate of measurement error and missed care associated with manual and intermittent vital signs monitoring methods (19–21).

As the number of wearable devices continues to grow, it is important to consider both health provider and patient perspectives (or parental perspectives in the case of newborns), and to evaluate the end-users’ experiences and opinions against the potential benefits that wearable devices might offer. Available evidence has shown that patient perspectives is essential as their acceptance of a new healthcare device influences its successful adoption and sustained utilization, ultimately impacting treatment outcomes and overall healthcare quality. We conducted a qualitative study to examine the acceptability, satisfaction and perceived benefits of neoGuard, an innovative wearable vital sign monitoring device, among nurses and parents/guardians (primarily mothers) of sick newborns in Nigeria. This paper described the experiences and perspectives gathered from the parents/guardians.

## Objectives

The objective of this study was to examine the acceptability, satisfaction, and perceived benefits of the neoGuard technology among the parents/guardians that consented to monitoring of their newborns with the novel device during their admission on a neonatal ward. This study was part of a series of feasibility pilot studies, conducted across a few countries.

## Materials and methods

### Design and setting

In this qualitative study, researchers conducted in-depth interviews to examine parental perceptions on the adoption of a wireless vital signs monitor for observation of sick newborns in low-resource settings. This qualitative study was part of a feasibility pilot study to evaluate the feasibility of using NeoGuard as a wireless monitor in three selected hospitals in Nigeria; to provide insights into the potential impact of neoGuard^TM^ on newborn health, and to generate preliminary efficacy data to inform sample size calculations for future effectiveness studies. The neoGuard technology was implemented at three health facilities in Nigeria between April and December 2022. The health facilities included University College Hospital (UCH) Ibadan, Oyo State (Site 1); Mother and Child Hospital (MCH), Akure, Ondo State (Site 2); and University of Medical Science Teaching Hospital (UNIMETH), Akure, Ondo State (Site 3). Study sites have been purposively selected based on geography, availability of a neonatal ward, high neonatal caseloads, low nurse-to-patient ratios and need for vital sign monitoring equipment.

The study received scientific and ethical approval from University of Ibadan/University College Hospital Ethics Committee (Number UI/EC/21/0243).

### Neoguard device

**Figure 1:**
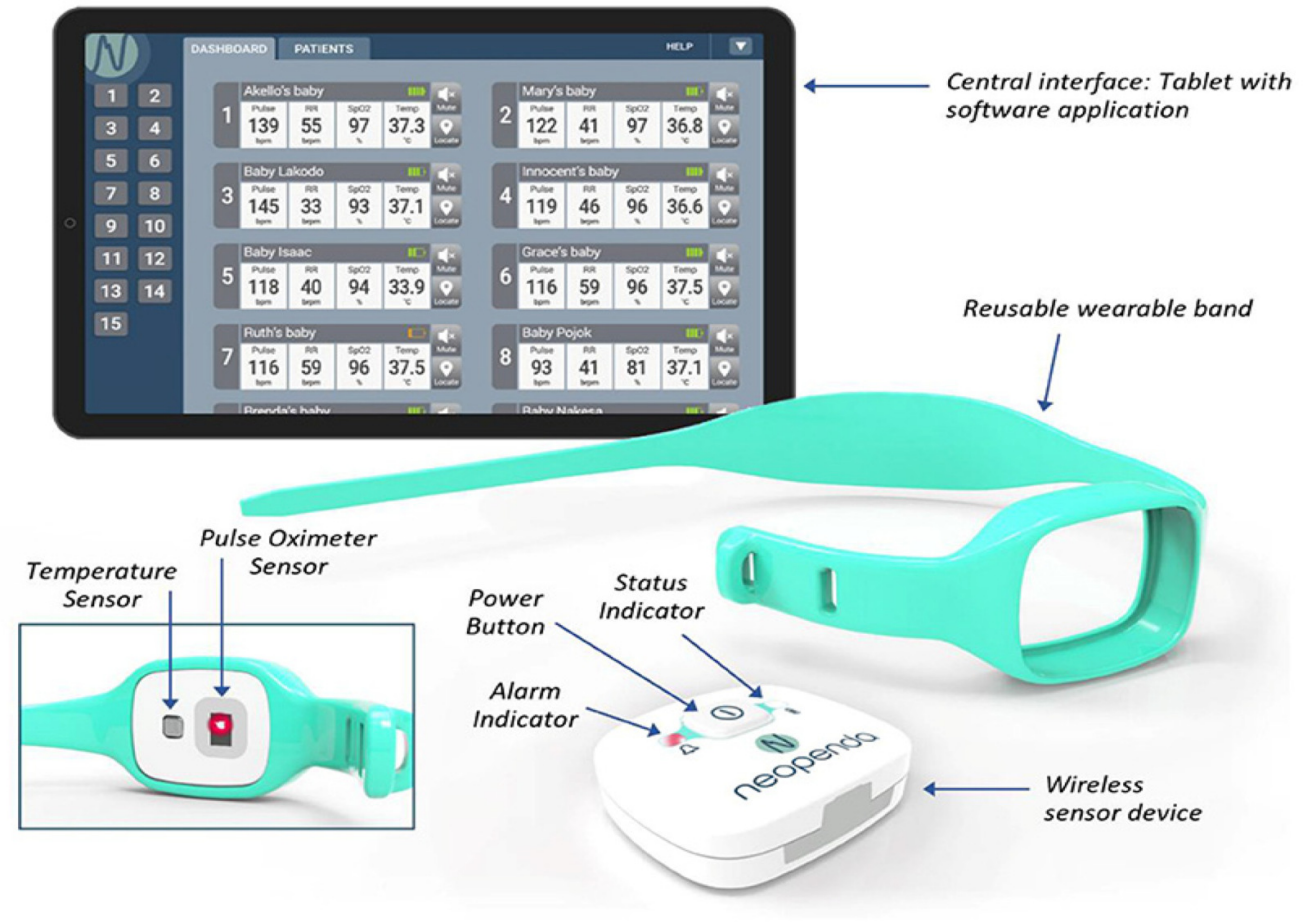
The neoGuard vital signs monitoring system. Reprinted with permission from Neopenda, original image created by Neopenda PBC, 2024.

The neoGuard technology is a wireless wearable vital signs monitor which continuously measures four vital signs: temperature, pulse rate, respiratory rate, and SpO2, transmitting readings to a centralized monitor on a tablet. The technology is intended to aid in early detection of deterioration in patients by generating real-time audio and visual alerts to notify health care providers when patient vital signs fall outside the expected normal range. The device consists of the following components: a wireless sensor, reusable wearable bands and a software application that receives, displays, and stores data from the sensor devices (Figure 1). The neoGuard device uses reflectance pulse oximeter and temperature sensors to measure vital signs from the patient, and the readings are displayed on a portable tablet screen, which can show vital signs for up to 15 patients simultaneously. It is battery powered with a long-lasting rechargeable battery. The device makes use of infrared light and sensors that measure the vital signs by picking up vital signs when placed over the skin. The technology is somewhat like what we have in pulse oximeters used in standard care. This is however unique, in that a separate temperature sensor is added. It also measures blood pressure, and all parameters measured can be transmitted wirelessly and synced on a tablet, making central monitoring easy. The device does not aim to replace physical face-to-face assessments, as those are still very necessary. However, it can help to quickly identify the clinical states of the newborns at a glance and pick up abnormalities immediately they occur, even before there are obvious clinical signs (see Supplementary Appendix for Flowchart).

The device was developed and provided by Neopenda, PBC (Chicago, IL, USA|Kampala, Uganda) under international medical device standards and for feasibility in low-resource settings. Initial training was conducted by the providers of the device, for the team facilitators (this included doctors and nurses) via a series of step-by-step sessions breaking down both technical and end-user information. The knowledge was then passed down to the end users across the 3 sites in Nigeria, who were continuously trained over time hands-on with the use of the equipment. Regular neonatal staff from each facility received training on how to use the neoGuard^TM^ technology. Following training, 10 neoGuard^TM^ devices and 1 central monitoring tablet were introduced to each neonatal unit. Consecutive admissions of eligible newborns were enrolled and monitored with the neoGuard^TM^ technology. The devices were worn for as long They needed continuous monitoring of vital signs. There were some breaks when the child wants to feed, during a procedure and so on. Skin pigmentation might not necessarily be a problem as better grip on skin (in this case the forehead of babies) have more guarantee of ensuring more accurate readings.

#### Mitigating potential long-term effects

While the neoGuard's primary goal is to prevent negative long-term outcomes by enabling timely care, its design also addresses common concerns associated with neonatal monitoring:

1. **Skin irritation:** Its non-adhesive, reusable design minimizes skin issues.
2. **Discomfort:** The compact, wireless nature reduces interference with care and bonding.
3. **False alarms:** Advanced algorithms and training help distinguish true emergencies from minor fluctuations.
4. **Accuracy:** Rigorous testing ensures reliable readings.
5. **Over-reliance on the device:** Comprehensive training emphasizes that the neoGuard is a tool to aid clinical judgment, not replace it.

By focusing on these areas through continuous improvement, the neoGuard strives to be a highly viable and impactful solution for improving newborn health outcomes.

#### Comparison with existing technologies

The neoGuard Vital Signs Monitor represents a significant leap forward in neonatal care, particularly when compared to traditional monitoring technologies and even some earlier attempts at wearable solutions. Its improvements primarily stem from a design philosophy centered on the unique challenges of low-resource settings and the fragility of neonates. The comparison and breakdown of the improvements include:

1. **Traditional Hospital Monitors (Wired, Multi-Parameter Monitors):**
  • **Existing Technology:** These are the large, often wheeled, bedside monitors found in NICUs in high-resource settings. They use multiple wired sensors (ECG electrodes for heart rate, separate pulse oximeter probes for SpO2, adhesive patches or rectal probes for temperature, and often impedance pneumography for respiratory rate).

**Disadvantages:**

○ **High Cost:** Very expensive to purchase, maintain, and replace parts, making them inaccessible for many low-resource hospitals.
○ **Infrastructure Dependent:** Require stable power supply, often air conditioning (for electronics longevity), and specific technical expertise for maintenance. These are frequently lacking in resource-constrained areas.
○ **Bulky and Immobile:** Not designed for portability or for monitoring multiple patients centrally without dedicated space.
○ **Invasive/Disruptive:** Multiple wires may entangle the baby, restrict movement, interfere with kangaroo care (skin-to-skin contact), and cause parental anxiety. Adhesive electrodes can lead to skin irritation, injury, and infection on delicate neonatal skin.
○ **Limited Central Monitoring:** Often, each monitor is tied to a single patient, making it difficult for limited staff to oversee many babies simultaneously.
○ **Designed for Adults:** Many sensors and parameters are adapted from adult monitoring, which may not be optimal for neonates with different physiological ranges and skin sensitivities.

2. **Basic Intermittent Monitoring (Manual or Single-Parameter Devices):**
  • **Existing Technology:** In very low-resource settings, monitoring might involve manual counting of breaths and pulse, or sharing a single, basic pulse oximeter used intermittently. Temperature might be taken with a standard thermometer.

**Disadvantages:**

○ **Lack of Continuity:** Intermittent checks may miss rapid deteriorations in a neonate's condition, which can change very quickly.
○ **Human Error:** Manual measurements are prone to inaccuracies and omissions.
○ **High Workload for Staff:** Requires constant attention from nurses, diverting them from other critical care tasks.
○ **Delayed Intervention:** Critical changes may go unnoticed for hours.

3. **Other Wearable/Home Monitors:**
  • **Existing Technology:** A growing market of wearable baby monitors exists, often for home use by parents (e.g., smart socks, clip-on devices).

**Disadvantages:**

○ **Clinical Validation:** Many lack rigorous clinical validation for medical-grade accuracy, especially for sick neonates.
○ **False Alarms:** Can generate frequent false alarms, causing undue parental anxiety and unnecessary emergency room visits.
○ **Limited Parameters:** May not monitor all crucial vital signs (e.g., some only track heart rate and oxygen).
○ **Not Designed for Clinical Workflow:** Typically not designed for multi-patient monitoring or integration into hospital systems.

**Improvements offered by neoGuard:**

The neoGuard aims to address the shortcomings of existing technologies with several key improvements:

1. **Tailored for Low-Resource Settings:**
  1. **Low Cost:** Designed from the ground up to be affordable for purchase and maintenance.
  2. **Robust and Self-Sufficient:** Operates reliably in environments with unstable power, limited internet, and high heat/humidity. Its long-lasting rechargeable battery (several days of use) is a huge advantage where continuous power is an issue.
  3. **Easy to Maintain:** Simple design and reusability reduce the need for expensive consumables and spare parts.
2. **Continuous, Multi-Parameter Monitoring:**
  4. **Comprehensive Data:** Simultaneously measures four critical vital signs (PR, RR, SpO2, Temperature), providing a holistic view of the neonate's condition.
  5. **Early Detection:** Continuous monitoring allows for the immediate detection of subtle changes or rapid deterioration, enabling swift intervention and potentially saving lives.
3. **Wearable, Non-Invasive, and User-Friendly Design:**
  6. **Comfort and Safety:** Worn on the forehead with a soft band, it minimizes skin irritation and avoids the discomfort and potential injury associated with adhesive electrodes or wired probes. This is crucial for fragile neonatal skin.
  7. **Promotes Bonding:** Its wireless and unobtrusive nature allows for easier skin-to-skin contact (kangaroo care) and parent-infant interaction, which is vital for neonatal development.
  8. **Simplified Application:** Easy to apply and remove, reducing the burden on nursing staff.
4. **Centralized, Intelligent Monitoring System:**
  9. **Tablet-Based Dashboard:** A single tablet can monitor up to 15 patients simultaneously, a game-changer for facilities with high patient-to-nurse ratios.
  10. **Real-Time Alerts:** Provides immediate visual and auditory alarms for abnormal vital signs, prioritizing urgent cases.
  11. **Data Archiving and Trends:** Stores historical data, allowing clinicians to track patient progress over time, which supports better clinical decision-making and informs treatment plans. This digitizes record-keeping, reducing human error.

The neoGuard, targeted for low resource settings, re-imagines neonatal vital signs monitoring, by providing a less costly, bulky, and traditional systems, to a user-friendly, affordable, reliable, and clinically effective wearable solution specifically designed to meet the unmet needs of vulnerable newborns in the most challenging healthcare environments.

### Sampling procedure

Our participants included parents/guardians of sick newborns who gave consent and their newborns were among those being monitored with the neoGuard technology. Newborns monitored were selected based on the criteria of age <28 days, admission to the NBCU/NICU, birth weight at admission ≥2,000 g, low-to-moderate disease severity as determined by the Signs of Inflammation in Children that Kill (SICK) score card (SICK score ≤2.5 at admission) (22) and parent/guardians willing to provide informed consent for their newborn to participate in study. Newborns were excluded from monitoring if they had a contraindicated condition like hydrocephalus or were receiving medicine through intravenous access via scalp veins. Parents/guardian were then selected based on the following criteria: parent/guardian whose newborn was enrolled, newborn completed monitoring and guardian who was present to observe at least 2 h of monitoring of their newborn with the neoGuard technology. For every 10 newborns enrolled into the study, one parent/guardian was selected by stratified random sampling to give a short interview. Information on use and benefits of the device was provided to each selected parent/guardian. They were interviewed using semi-structured interview guides to gain insights on their perception and acceptability of the device upon experience. Interviews were conducted in each participant's preferred language.

A conceptual framework was adapted from Asiimwe et al. (23) to inform the design of the qualitative interviews. Elements on willingness, suitability, satisfaction and likeability were embedded into the qualitative interviews to evaluate the acceptability, satisfaction and perceived benefits of using NeoGuard from parents/guardians’ perspectives. This framework was adapted to offer insights into the dynamics of health behaviour regarding use of the new technology (neoGuard) and processes that could be employed in effecting changes during the implementation process. The attributes measured work in an interrelated way with the meanings below:

1. *Willingness*: Parents/guardians’ willingness to accept the NeoGuard use for their neonates
2. *Suitability*: Parents/guardians’ belief that NeoGuard is suitable for monitoring their baby's vital signs
3. *Satisfaction*: Parents/guardians’ satisfaction with the way technology is used on their baby and benefit to newborn health
4. *Likeability*: Parents/guardians’ like and are happy with the NeoGuard technology, and is pleasing to them for use and perceived as beneficial to their baby.

### Data collection

All interviews were conducted by trained research assistants and moderated by a semi-structured interview guide. Before the interview, the research assistants obtained written informed consent from each parent/guardian interviewed. Each interview was conducted in a private setting preferred and lasted approximately 25 min. A Sony recorder was used to record the discussions and recordings were transcribed verbatim. Notes taken were used to supplement the analysis of transcripts. Of the 23 parents/guardians sampled, 20 consented and participated in an in-depth interview. The field exercise ended when saturation was reached and no new concept emerged.

### Data analysis

Recorded voices and notes taken were transcribed verbatim and word-processed. For confidentiality, “*P1, 2, 3…*” and “*I*” were used to denote the names and identities of participants/interviewers. Where applicable, “xxx” was introduced to eliminate any identifiers relating to the status of participants, location, position, and so on. NVivo QSR version 12.2 Pro software was used for the analysis. Thematic analysis through inductive and deductive approaches was used.

Subsequently, there was a revision of the codes which were labeled and described, and thereafter, a line-by-line coding of all the transcripts using the descriptive consolidated criteria for reporting qualitative research (COREQ) by Tong et al. (24). Codes were developed based on the pre-existing guides and new themes that emerged from transcripts. The approach started with a predefined set of codes designed for the research questions. A pre-codebook was developed, scrutinized, and agreed upon by qualitative analyst before using it to develop further codes. Contextual and denotative cross-checking was carried out to identify wrongly assigned codes; non-conforming codes were removed and too-long codes were redefined appropriately. These codes were then assigned to the transcripts based on themes and sub-themes. These themes/sub-themes were later accrued to form the themes used for the analysis and reporting.

## Results

### Socio demographic characteristics of participants

The majority (17 of 20) of the participants were mothers of newborns with a mean (SD) age of 29.3 years. Many (40%) were traders; 15% were fashion designers; with the least being hair stylist, auto-mechanic, and entrepreneur. Less than half of participants (45%) hold senior secondary school certificates, with 30% having NCE/ND as their highest educational attainment. Because the study was conducted in Southwest Nigeria, 80% were Yoruba indigenes, with 10% being Igbo and Ebira respectively. The highest age of the participants ranged between 20 and 29 years while that of their babies was between 0 and 9 days. On average, the device was used on these babies between 3 and 5 days (Table 1).

**Table 1:** Participants’ socio-demographics characteristics (*N* = 20).

Demographics	Frequency	%
Status of participants		
Mother of the child	17	85
Father of the child	2	10
Aunt of the child	1	5
Occupation/Profession		
Trader	8	40
Teacher	2	10
Unemployed/student	2	10
Mechanic	1	5
Fashion Designer	3	15
Hairstylist	1	5
Auxiliary nurse	2	10
Entrepreneur	1	5
Educational level		
No formal Education	1	5
SSCE	9	45
Diploma (NCE/ND)	6	30
Bachelor/HND	4	20
Ethnic Group		
Yoruba	16	80
Igbo	2	10
Hausa	0	0
Others (Ebira)	2	10
Age as at the last birthday (in years)		
17–19	2	10
20–29	10	50
30–39	5	25
40–49	2	10
50–59	1	5
Age of the baby (in days)		
0–9	8	40
10–20	6	30
21–30	2	10
31–40	1	5
41–above	3	15
The observed number of days of use of the device		
0–2	8	40
3–5	12	60

### Acceptability of the neoGuard vital signs monitoring

Reactions of participants to the device varied, however, with majority positive feelings. Despite consenting to participate in the study, the majority reported being amazed at sighting the “*electric band*”. These reactions necessitated more questions, the quest to know what it does, and a need to “*feel*” the device by touching or moving closer during use. Four groups of reactions were observed and documented; (1) some were scared and surprised seeing the device, (2) some could not express their feelings, for others, (3) seeing the device made them inquire and search online for its usefulness and to many others, (4) no reaction because they have been pre-informed:

“When he (the doctor) first talk to me about it, I was scared because of all the explanations, based on the condition of the baby when I took him to the hospital (hissed) so I was like ‘is it part of the test I need to do’, so you understand. So I was I was calm, but when I read the paper he gave to me, I saw nothing bad in it”**<P5, Site 1>**.

“Nothing. There was no reaction since it is meant for treatment…” **<P1, Site 3>**.

On the contrary, two of the participants reported not being comfortable with the use of the device on the forehead of their babies, hence, they reported to have *reluctantly* allowed it. Nevertheless, the outcomes of the use of the device negated their assertions: As narrated:

“Me, my concern is that the baby should be comfortable with the thing. So, when the thing was introduced and that thing will need to touch the baby’s forehead with no cap touching his head…so I was not comfortable with it but later on, when an extension was added to it so, it went well (I: so, but what was reaction for seeing that device itself) Me I was like ‘I don’t want to since they have something to measure the temperature before. But when a further explanation was made, I just had to comply that they should use it for that period” (I: why do you just have to comply?)…But later when an explanation was made, so, I don’t know, that’s all”**<P6, Site 1>**.

While some were still doubting its functions, others were impressed with the device:

“I was so impressed, I was happy, because it was developed for use on children. I knew that it will encourage most people well…I saw it and how it reads on the child…. And the readings correspond with all they are doing”**<P8, Site 1>**.

‘Feelings’ expressed at the attachment of the device to their baby’s head were similar to that expressed when it was first seen by participants. However, there were variations in these expressions. To some, seeing the device attached on their baby’s head created a funny scene and they loved to have it used perpetually: “Yes now. Hahaha (burst into laughter) because even if I see a bigger one, I will put it on my head (burst into laughter again)”<P2, Site 3>.

To one participant, it was a sign that the baby was undergoing a serious health challenge, hence, not comfortable, “*I was not comfortable actually because there must be something wrong if you see this kind of thing in your baby’s head. So, I did not feel comfortable. I was worried because I don’t know what is happening to my baby that… I was overwhelmed, I was concerned…I don’t like seeing little babies with all those things. It gives me like this fear…*”***<P1, Site 1>****.*

However, some other participants were concerned with the use of the device on babies’ forehead because of the fear of side effects. This was repeatedly expressed by these participants during the study, and many suggested the use of wrist bands instead.

Emerging from the discussions was the level of confidence in the health workers, which made participants permit the testing of the device on their babies. This level of “*confidence*” was attributed to the trust and relationship developed with service providers. To many of them, “*(the) health worker and government meant good by allowing the study, as such, they cannot allow or permit harm to children*, hence the assertions ‘*I know my baby is safe and they (health workers) cannot harm my baby*’”. Almost all appeared to be “very” confident of the safety of their babies in this study, as such, confidence, relationship, professionalism and the type of health facility made them to participate in the study. To some others, the relationship built with health workers also necessitated and earned their “*trust*”, hence, the reason for permitting the use of the device on and for their children as documented:

“(I: Why weren’t you afraid?) I know it cannot harm my baby (I: why)…we were simultaneously watching and seeing the reading through the computer device that looks like laptop…that was why I was not afraid”**<P1, Site 2>**. “I know that my baby is safe with this”**<P1, Site 1>**

“Ehn….because they are people that I'm familiar with…it’s not the first time they are attending to me, they’ve attended to me even before the baby was delivered…I have spoken with them, I have met them, I saw them, so I think there is a…..we already have a relationship, I already have a relationship with them which is cool by me. And I think I was comfortable with them using it for her (my baby)”**<P4, Site 3>**.

### Satisfaction of the neoGuard vital signs monitoring

Feelings of satisfaction documented were based on the perceived benefits and expressed fears/dangers. Some expressed their satisfaction enthusiastically. Others were not favorably disposed, hence, not fully satisfied. These they attributed to the use of the device on the forehead as documented from few participants:

“Yes (I: How) because when the thing was placed on her head, is not that the device is disturbing her or she is reacting anyhow, she is sleeping, she wasn’t even giving any sign as if something was on her head, so she (stutters) she didn’t give any negative reaction to it. Like crying or anything, so she slept without any negative reaction, so I see no bad in it…the doctor did not just come often to wake the baby”**<P5, Site 1>**.

“Yes. The only reservation I had was ehn…..the use on the head. Hmm….I was like (coughs) why not on the hand? Why (why) the head? So, that was the only reservation (reservations) I (I) had… I just have issues with it on the baby’s head. You know ehmm…. You wrapping it and you have to…. Fit the baby’s head but ehn you know I just thought may be the child might not be comfortable and you know the baby will not be able to talk and ehn….. You know there is no how they won't touch the baby’s head. So, I just felt why not on the hand?”**<P4, Site 3>**.

All participants reported noninterference of the device during cleaning, breastfeeding, kangaroo care, however, evidence from transcripts showed that the device was removed from many babies’ head during these processes: this was done to ensure *correct readings and prevent damaging*. As summarized in these excerpts: “*It did not interfere because it wasn’t that long at all. I breastfeed the baby at the usual time and I laid him to rest and sleep, so it didn’t interfere. When I was breastfeeding, it was placed on him and it was not too tight nor did it disturb him or me. It didn’t disturb me in cleaning, breast feeding or doing kangaroo care*”**<P1, Site 2>**.

Participants opined that the device would contribute positively to the care of the newborn. In this case, no bad or negative notion was raised. These excerpts summarised their thoughts:

“*Yes, it will contribute positively because you might be busy; nurse might be busy doing something and your mind is not where the baby is; but when you hear that sound (of the device), you will be tracing where is that sound coming from. That thing will lead you to know that something is happening to that baby… to go and check the baby that something, danger (danger)…”**<P3, Site 1>**.*

### Perceived benefits of neoGuard vital signs monitoring

Many benefits were accrued to the functions of the device; of these, few mentioned its core functions (temperature, pulse rate, heart rate, respiratory rate). This led to the coding of this emerging theme, as many resonated its functions to include magnetic resonance imaging (MRI), echocardiogram, “magnetic healing” machine, baby sleep soothers, internal diagnosing machines, ultrasound device, etc. These were attributed to the outcomes of its use on their wards/children. The below excerpts described these assertions:

“(Laughed in amazement at the question) Number 1, I am so happy because if something is wrong with the baby somewhere before now, it is possible for this device to detect it. Then, in fact, that alone is enough. You know, the device will unravel all things that are yet to be known to us…This will help know the necessary steps to take in order to cure such disease or ailment in a short while”**<P5, Site 2>**.

“…if something is wrong in his head/brain, the device will help correct it well. So, these were my positive thoughts about it. Again, you know some children are born, they may by accident, hurt or injured their head during the process. So, I felt the device is helping to check if there are complications so that we will know what is actually affecting the baby within his system…”**<P1, Site 2>**.

Related to the above is another emerged theme which explored whether the device will be useful to improve baby’s health if introduced into hospital system. This received good acknowledgment, with majority advocating for a quick introduction of the device. All participants opined that the device may be suitable for monitoring baby’s vital signs provided it is designed to carry out the ascribed functions. In this sense, size and shape does not matter. Guardians were therefore ready to permit concurrent use of the device on their wards/children if and when requested for follow-up.

“Yeah, why not? If it will be the same result as the manual ehn…the manual (the manual) gadgets, If we will get the same result, why not?…we have issues with parents not bringing their thermometer, their babies digital thermometer to the hospital…For the manual it will take you 15 min, this one will take you 5 min for instance so in a way it’s saving you time and less stressful. You know at times maybe you want to check temperature; you have to pull some babies clothes. So, it is more convenient for you to use…The handler, if the handler is properly trained, why not? It’s a cool gadget why not. It should be introduced”**<P4, Site 3>**.

“Yes, yes. It is not because it is small or maybe it is big that is what will make it do the require something. The size doesn’t matter because as small as it, it can do whatever it is meant to do”**<P6, Site 2>**.

On whether any complaint was raised during its use on babies, the majority (18 of 20) did not raise or hear any complaints. However, the issue of how accurately the device measures what it is supposed to and use on the head were the two complaints documented by participants. Generally, all participants reported liking the device. Among what they liked included its indicator lights, size, shape, color, and functions:

“Yes, it was too tight for his head, and when I called their attention to it, it was adjusted”**<P7, Site 1>.**

“Altogether, the device is fine since it is used for health purposes. The color, the way the device beeps are okay”**<P3, Site 2>.**

“(smiles) Of course. I like it, it’s ehm… (what will I say). It’s beautiful and it’s simple for the baby; it doesn’t discomfort the baby and it’s fashionable…Hmm hmm…”**<P4, Site 1>.**

However, most participants preferred to see the device affixed on the wrist rather than the head. As explained:

“…it draws line. Yes, it leaves mark on his head. It separates his head and the device is long. The way it is long is what I dislike. If shortened, it will be fashionable and it will be more presentable than this. It should be made into wristwatch-like device”**<P2, Site 3>**

“…what I just dislike is because it is too big on the baby head; they (makers) should help us to reduce it”**<P3, Site 3>**.

## Discussion

Parents play a considerable role in providing supportive care to hospitalized newborns. They are actively involved in many critical aspects of patient management including feeding, cleaning, and reporting of observed symptoms to health staff. This is especially the case on neonatal units in low-resource settings, where due to limited ward staff, parental contact becomes essential to bridging the gap in nurse-patient contact for critically ill newborns (25). Parental perceptions on any new procedures or technology intended to improve neonatal care are therefore incredibly valuable to the teams implementing them, as parents will observe and interact with these systems at a high frequency as they care for their newborn. Understanding the concerns and anxieties of parents is crucial if the solution is to be successfully adopted and achieve the desired impact.

Our study was part of a pilot study and reported the qualitative aspect, which sought to explore the perceptions and experience of the parents/guardians who observed the use of the neoGuard vital signs monitor on their sick newborns. Currently, limited studies have been conducted in a similar setting of acute patients (26) and in particular, critically sick neonates in resource limited areas (27, 28). Our technology is therefore a pioneer in exploring parental acceptability and satisfaction of wearable vital sign monitoring devices in such a setting.

Overall, the neoGuard technology was viewed positively by the parents/guardians interviewed. Similar to other studies exploring patient perceptions and acceptability, a majority of our participants noted perceived benefits of functionality as the device ensured timely detection of deterioration of health, is time saving and easy to use to monitor sick patients (10, 11, 13).

The general design, colour, size and shape of the neoGuard device was acceptable to a majority of our participants. However, a few participants mentioned preferring the device to be designed to monitor vitals from the wrist instead of around the forehead. Two parents expressed concern of having their newborns monitored using the technology. This was majorly due to perceived discomfort with placement of the device on the forehead and the tight grip of the technology on the newborns. These were user related challenges that were mitigated by refresher trainings and illustrations. One participant also expressed their fear of thinking their newborn was having a serious health challenge to warrant monitoring using our technology.

It is common for patients or parents of patients to feel skeptical about new medical devices on first contact (27). However, similar studies have reported that these fears and doubts can be easily alleviated by giving accurate information through education on the device, what it does and how it functions prior to monitoring patients (13). Further still, users of these technologies have expressed confidence in users who have been trained on use of wireless vital signs monitoring technology. Findings from a mixed methods study by Leenen et al. revealed that medical practitioners reported better understanding of a wearable monitoring device with continuous on-job training and practice (10).

Our study had limitations of a small sample size for generalizability of findings. However, we surveyed participants from three different hospitals to ensure a wide range of responses and understanding of the experience of utilizers of the technology. That said, male participants were underrepresented in this study. With only 2 fathers being interviewed, the perspectives of fathers were not adequately explored and may be less generalizable to the broader community. One strength of our study was that we monitored patients (neonates) for up to five days per patient which we consider sufficient time for the parents/guardians who were interviewed to observe the technology and share insights based on deep understanding of the technology. Our study was also conducted among neonates and in a resource- limited setting, a largely unexplored population with wearable continuous vital signs monitoring technologies.

## Conclusion

This study sought to explore the perceptions and experience of parents/guardians who consented to the use of a novel wearable wireless vital signs monitor for observation of their hospitalized newborn. The neoGuard technology was generally acceptable to parents/guardians of newborns in this setting. Parents particularly liked the functionality, efficiency, and the effectiveness of the device, with feedback on areas to improve on such as design of the device to maximize patient comfort. As more wearable innovations are introduced in clinical practice, the adoption of these technologies will be dependent on user satisfaction and patient experience, as well as the experiences of guardians like parents for vulnerable patient groups. The feedback from these groups will go a long way towards ensuring a refined final product that delivers the most value. This study generated important parental insights from the early implementation of a wearable continuous vital signs monitor in a low-resource newborn care setting. Future studies examining areas like performance, clinical impact, or cost-effectiveness of wearable devices should simultaneously weigh those outcomes against the perceptions and experiences of the health staff, patients monitored, and/or any guardians who will interface with the technology.

## Data Availability

The original contributions presented in the study are included in the article/Supplementary Material, further inquiries can be directed to the corresponding author.
