# Supplementary material for: Parental perceptions of a wearable vital signs monitor for observation of newborns: in-depth interviews in three tertiary hospitals, Southwest Nigeria
Source: Front Digit Health. 2025 Jul 28;7:1597651. doi: 10.3389/fdgth.2025.1597651 (PMC12337009; doi:10.3389/fdgth.2025.1597651)
Supplement: Supplementary file 1 [file Datasheet1.pdf]

SUPPLEMENTARY MATERIAL

A. Flowchart: How NeoGuard Works

Start device

NeoGuard Device Placement (Forehead)

Sensors Activated (HR, RR, SpO_2, Temp)

Data Processing in Device

Wireless Transmission via Bluetooth

Real-Time Display on Dashboard

Alert System Flags Abnormal Vitals

Clinical Decision by Caregiver

End and turn off device
